# 84 The Association between Family Relations and School Outcomes in School-Aged Children 5-12 years old

**DOI:** 10.1093/jbcr/irad045.058

**Published:** 2023-05-15

**Authors:** Madeleine McGwin, Khushbu Patel, Pengsheng Ni, Frederick Stoddard, Ludwik Branski, Petra Warner, Jeffrey Schneider, Lewis Kazis, Colleen Ryan, Tina Palmieri, Kate Surette

**Affiliations:** Shriners Children's- Boston, Boston, MA, Boston, Massachusetts; Shriners Children's Boston, Massachusetts General Hospital, Boston, Massachusetts; Boston University School of Public Health, Boston, Massachusetts; Harvard Medical School at the Massachusetts General Hospital, Boston, Massachusetts; Shriners Children's- Texas, University of Texas Medical Branch, Galveston, Texas; Shriners Children's Ohio, Dayton, Ohio; Spaulding Rehabilitation Hospital/Harvard Medical School/Rehabilitation Outcomes Center at Spaulding, Boston, Massachusetts; Boston University School of Public Health, Rehabilitation Outcomes Center at Spaulding, Boston, Massachusetts; Shriners Children's-Boston, Massachusetts General Hospital, Harvard Medical School, Boston, Massachusetts; UC Davis and Shriners Children's Northern California, Sacramento, California; Shriners Children's - Boston, Boston, Massachusetts; Shriners Children's- Boston, Boston, MA, Boston, Massachusetts; Shriners Children's Boston, Massachusetts General Hospital, Boston, Massachusetts; Boston University School of Public Health, Boston, Massachusetts; Harvard Medical School at the Massachusetts General Hospital, Boston, Massachusetts; Shriners Children's- Texas, University of Texas Medical Branch, Galveston, Texas; Shriners Children's Ohio, Dayton, Ohio; Spaulding Rehabilitation Hospital/Harvard Medical School/Rehabilitation Outcomes Center at Spaulding, Boston, Massachusetts; Boston University School of Public Health, Rehabilitation Outcomes Center at Spaulding, Boston, Massachusetts; Shriners Children's-Boston, Massachusetts General Hospital, Harvard Medical School, Boston, Massachusetts; UC Davis and Shriners Children's Northern California, Sacramento, California; Shriners Children's - Boston, Boston, Massachusetts; Shriners Children's- Boston, Boston, MA, Boston, Massachusetts; Shriners Children's Boston, Massachusetts General Hospital, Boston, Massachusetts; Boston University School of Public Health, Boston, Massachusetts; Harvard Medical School at the Massachusetts General Hospital, Boston, Massachusetts; Shriners Children's- Texas, University of Texas Medical Branch, Galveston, Texas; Shriners Children's Ohio, Dayton, Ohio; Spaulding Rehabilitation Hospital/Harvard Medical School/Rehabilitation Outcomes Center at Spaulding, Boston, Massachusetts; Boston University School of Public Health, Rehabilitation Outcomes Center at Spaulding, Boston, Massachusetts; Shriners Children's-Boston, Massachusetts General Hospital, Harvard Medical School, Boston, Massachusetts; UC Davis and Shriners Children's Northern California, Sacramento, California; Shriners Children's - Boston, Boston, Massachusetts; Shriners Children's- Boston, Boston, MA, Boston, Massachusetts; Shriners Children's Boston, Massachusetts General Hospital, Boston, Massachusetts; Boston University School of Public Health, Boston, Massachusetts; Harvard Medical School at the Massachusetts General Hospital, Boston, Massachusetts; Shriners Children's- Texas, University of Texas Medical Branch, Galveston, Texas; Shriners Children's Ohio, Dayton, Ohio; Spaulding Rehabilitation Hospital/Harvard Medical School/Rehabilitation Outcomes Center at Spaulding, Boston, Massachusetts; Boston University School of Public Health, Rehabilitation Outcomes Center at Spaulding, Boston, Massachusetts; Shriners Children's-Boston, Massachusetts General Hospital, Harvard Medical School, Boston, Massachusetts; UC Davis and Shriners Children's Northern California, Sacramento, California; Shriners Children's - Boston, Boston, Massachusetts; Shriners Children's- Boston, Boston, MA, Boston, Massachusetts; Shriners Children's Boston, Massachusetts General Hospital, Boston, Massachusetts; Boston University School of Public Health, Boston, Massachusetts; Harvard Medical School at the Massachusetts General Hospital, Boston, Massachusetts; Shriners Children's- Texas, University of Texas Medical Branch, Galveston, Texas; Shriners Children's Ohio, Dayton, Ohio; Spaulding Rehabilitation Hospital/Harvard Medical School/Rehabilitation Outcomes Center at Spaulding, Boston, Massachusetts; Boston University School of Public Health, Rehabilitation Outcomes Center at Spaulding, Boston, Massachusetts; Shriners Children's-Boston, Massachusetts General Hospital, Harvard Medical School, Boston, Massachusetts; UC Davis and Shriners Children's Northern California, Sacramento, California; Shriners Children's - Boston, Boston, Massachusetts; Shriners Children's- Boston, Boston, MA, Boston, Massachusetts; Shriners Children's Boston, Massachusetts General Hospital, Boston, Massachusetts; Boston University School of Public Health, Boston, Massachusetts; Harvard Medical School at the Massachusetts General Hospital, Boston, Massachusetts; Shriners Children's- Texas, University of Texas Medical Branch, Galveston, Texas; Shriners Children's Ohio, Dayton, Ohio; Spaulding Rehabilitation Hospital/Harvard Medical School/Rehabilitation Outcomes Center at Spaulding, Boston, Massachusetts; Boston University School of Public Health, Rehabilitation Outcomes Center at Spaulding, Boston, Massachusetts; Shriners Children's-Boston, Massachusetts General Hospital, Harvard Medical School, Boston, Massachusetts; UC Davis and Shriners Children's Northern California, Sacramento, California; Shriners Children's - Boston, Boston, Massachusetts; Shriners Children's- Boston, Boston, MA, Boston, Massachusetts; Shriners Children's Boston, Massachusetts General Hospital, Boston, Massachusetts; Boston University School of Public Health, Boston, Massachusetts; Harvard Medical School at the Massachusetts General Hospital, Boston, Massachusetts; Shriners Children's- Texas, University of Texas Medical Branch, Galveston, Texas; Shriners Children's Ohio, Dayton, Ohio; Spaulding Rehabilitation Hospital/Harvard Medical School/Rehabilitation Outcomes Center at Spaulding, Boston, Massachusetts; Boston University School of Public Health, Rehabilitation Outcomes Center at Spaulding, Boston, Massachusetts; Shriners Children's-Boston, Massachusetts General Hospital, Harvard Medical School, Boston, Massachusetts; UC Davis and Shriners Children's Northern California, Sacramento, California; Shriners Children's - Boston, Boston, Massachusetts; Shriners Children's- Boston, Boston, MA, Boston, Massachusetts; Shriners Children's Boston, Massachusetts General Hospital, Boston, Massachusetts; Boston University School of Public Health, Boston, Massachusetts; Harvard Medical School at the Massachusetts General Hospital, Boston, Massachusetts; Shriners Children's- Texas, University of Texas Medical Branch, Galveston, Texas; Shriners Children's Ohio, Dayton, Ohio; Spaulding Rehabilitation Hospital/Harvard Medical School/Rehabilitation Outcomes Center at Spaulding, Boston, Massachusetts; Boston University School of Public Health, Rehabilitation Outcomes Center at Spaulding, Boston, Massachusetts; Shriners Children's-Boston, Massachusetts General Hospital, Harvard Medical School, Boston, Massachusetts; UC Davis and Shriners Children's Northern California, Sacramento, California; Shriners Children's - Boston, Boston, Massachusetts; Shriners Children's- Boston, Boston, MA, Boston, Massachusetts; Shriners Children's Boston, Massachusetts General Hospital, Boston, Massachusetts; Boston University School of Public Health, Boston, Massachusetts; Harvard Medical School at the Massachusetts General Hospital, Boston, Massachusetts; Shriners Children's- Texas, University of Texas Medical Branch, Galveston, Texas; Shriners Children's Ohio, Dayton, Ohio; Spaulding Rehabilitation Hospital/Harvard Medical School/Rehabilitation Outcomes Center at Spaulding, Boston, Massachusetts; Boston University School of Public Health, Rehabilitation Outcomes Center at Spaulding, Boston, Massachusetts; Shriners Children's-Boston, Massachusetts General Hospital, Harvard Medical School, Boston, Massachusetts; UC Davis and Shriners Children's Northern California, Sacramento, California; Shriners Children's - Boston, Boston, Massachusetts; Shriners Children's- Boston, Boston, MA, Boston, Massachusetts; Shriners Children's Boston, Massachusetts General Hospital, Boston, Massachusetts; Boston University School of Public Health, Boston, Massachusetts; Harvard Medical School at the Massachusetts General Hospital, Boston, Massachusetts; Shriners Children's- Texas, University of Texas Medical Branch, Galveston, Texas; Shriners Children's Ohio, Dayton, Ohio; Spaulding Rehabilitation Hospital/Harvard Medical School/Rehabilitation Outcomes Center at Spaulding, Boston, Massachusetts; Boston University School of Public Health, Rehabilitation Outcomes Center at Spaulding, Boston, Massachusetts; Shriners Children's-Boston, Massachusetts General Hospital, Harvard Medical School, Boston, Massachusetts; UC Davis and Shriners Children's Northern California, Sacramento, California; Shriners Children's - Boston, Boston, Massachusetts; Shriners Children's- Boston, Boston, MA, Boston, Massachusetts; Shriners Children's Boston, Massachusetts General Hospital, Boston, Massachusetts; Boston University School of Public Health, Boston, Massachusetts; Harvard Medical School at the Massachusetts General Hospital, Boston, Massachusetts; Shriners Children's- Texas, University of Texas Medical Branch, Galveston, Texas; Shriners Children's Ohio, Dayton, Ohio; Spaulding Rehabilitation Hospital/Harvard Medical School/Rehabilitation Outcomes Center at Spaulding, Boston, Massachusetts; Boston University School of Public Health, Rehabilitation Outcomes Center at Spaulding, Boston, Massachusetts; Shriners Children's-Boston, Massachusetts General Hospital, Harvard Medical School, Boston, Massachusetts; UC Davis and Shriners Children's Northern California, Sacramento, California; Shriners Children's - Boston, Boston, Massachusetts

## Abstract

**Introduction:**

School-aged children between the ages of 5 and 12 years face new environments and experiences as they transition through grade school. They are still developing and learning as they engage within their home and carry these experiences into their school environment. This study is an assessment of the association between family relations and school outcomes in children 5 to 12 years of age as measured by the field-tested SA-LIBRE_5-12_ (School-Aged Life Impact Burn Recovery Evaluation), a new parent-reported outcome measure.

**Methods:**

Within the social and family functioning domain of the SA-LIBRE_5-12_, 10 family relations and 13 school items were scored on a scale from 0 (Never) to 4 (Always). Scores were re-coded so that higher scores denote better functioning. An exploratory factor analysis (EFA) was conducted to confirm model fit. A regression model assessed the relationship between the school and family factors, controlling for demographic and clinical characteristics (child sex, race, ethnicity, age at survey completion, burn size, burns to critical areas, and pain severity).

**Results:**

A total of 356 parents completed these items from the SA-LIBRE_5-12_. EFA resulted in four sub domains of family engagement, family stress impact, school success, and school difficulties. A univariate analysis of the demographic characteristics showed that the child being female (*p=*0.008) and non-white (*p=*0.0216) had a better mean score in school success (3.33 vs. 3.19 + 0.78 (SD) and 3.38 vs. 3.21 + 0.75 (SD), respectively). In addition, children with a burn size < 1% had better school difficulties score (3.76 vs 3.61 + 0.38, *p=*0.0018). Regression analysis showed positive correlations between family engagement and school difficulties (*r=*0.26, *p*< .0001), family engagement and school success (*r* = 0.56, *p*< .0001), family stress impact and school success (*r=*0.21, *p*< .0001) and family stress impact and school difficulties (*r=*0.49, *p*< .0001).

**Conclusions:**

This study indicates that a cohesive family relationship and home life may promote productive school outcomes in children between the ages of 5 and 12 based on parent reported outcomes.

**Applicability of Research to Practice:**

This research will provide burn care providers, parents, and teachers with greater insight into the relationship between a child’s current family relations and their success in school. Results will guide caregivers in fostering a productive and healthy family environment.

